# Tracking the Source of Human Q Fever from a Southern French Village: Sentinel Animals and Environmental Reservoir

**DOI:** 10.3390/microorganisms11041016

**Published:** 2023-04-13

**Authors:** Younes Laidoudi, Elodie Rousset, Anne-Sophie Dessimoulie, Myriam Prigent, Alizée Raptopoulo, Quentin Huteau, Elisabeth Chabbert, Catherine Navarro, Pierre-Edouard Fournier, Bernard Davoust

**Affiliations:** 1 Aix Marseille University, IRD, AP-HM, MEPHI, 13005 Marseille, France; 2 IHU Méditerranée Infection, 13005 Marseille, France; 3 ANSES, Laboratoire de Sophia Antipolis, Unité fièvre Q animale, 06902 Sophia Antipolis, France; 4 Clinique Vétérinaire des 4 Chemins, 34110 Vic-la-Gardiole, France; 5 Laboratoire d’Analyses Médicales Biomed 34, 34110 Mireval, France; 6 Cabinet Médical, 34110 Vic-la-Gardiole, France; 7 Aix Marseille University, IRD, AP-HM, SSA, VITROME, 13005 Marseille, France; 8 Centre National de Référence Rickettsies, Bartonella et Coxiella, 13005 Marseille, France

**Keywords:** One Health, *Coxiella burnetii*, Q fever, sentinels, epidemiology

## Abstract

*Coxiella burnetii*, also known as the causal agent of Q fever, is a zoonotic pathogen infecting humans and several animal species. Here, we investigated the epidemiological context of *C. burnetii* from an area in the Hérault department in southern France, using the One Health paradigm. In total, 13 human cases of Q fever were diagnosed over the last three years in an area comprising four villages. Serological and molecular investigations conducted on the representative animal population, as well as wind data, indicated that some of the recent cases are likely to have originated from a sheepfold, which revealed bacterial contamination and a seroprevalence of 47.6%. However, the clear-cut origin of human cases cannot be ruled out in the absence of molecular data from the patients. Multi-spacer typing based on dual barcoding nanopore sequencing highlighted the occurrence of a new genotype of *C. burnetii*. In addition, the environmental contamination appeared to be widespread across a perimeter of 6 km due to local wind activity, according to the seroprevalence detected in dogs (12.6%) and horses (8.49%) in the surrounding populations. These findings were helpful in describing the extent of the exposed area and thus supporting the use of dogs and horses as valuable sentinel indicators for monitoring Q fever. The present data clearly highlighted that the epidemiological surveillance of Q fever should be reinforced and improved.

## 1. Introduction

Q fever is a worldwide episystem-related disease, wherein the epidemiological features vary according to geographic area, including situations where it is endemic or hyperendemic and the occurrence of an epidemic breaks out. Q fever was originally described by Derrick in 1937 as a zoonotic disease caused by an intracellular bacterium, namely *Coxiella burnetii* [1,2]. Clinical symptoms encompassing reproductive disorders are described in ruminants. Abortion, stillbirth, and weak offspring are clearly associated with Q fever, while infertility and metritis have been suggested [3,4]. In humans, Q fever is characterised by acute forms with an influenza-like syndrome, pneumonia, and hepatitis, as well as by post-fatigue syndrome or persistent, localised infections, mainly with endocarditis and vascular infections [5]. Consequently, Q fever is involved in a significant public health problem [6,7,8].

Human infection mainly occurs in the vicinity of infected ruminants (i.e., sheep, goats, and, less frequently, cattle), but the role of other domestic and wildlife species as a source of *C. burnetii* infection seems minor but should not be overlooked during epidemiological surveillance [9]. The main transmission route remains the inhalation of aerosols contaminated with *C. burnetii* shed through domestic ruminants with birth products (vaginal secretions, placentas, aborted foetus) or faeces from infected ruminants [7,8]. The risk of infection via the oral route is still under discussion, but available investigations indicate that the consumption of dairy products from infected animals may lead to seroconversion but no clinical manifestation [10,11,12]. Due to airborne transmission, the high potential environmental survival, and the risk of causing long-term persistent infections [6], *C. burnetii* is classified by the Centers for Disease Control and Prevention (USA) as a potential agent of bioterrorism, which has resulted in the disease becoming reportable in many countries [1].

Measures involving the One Health paradigm represent a strong framework for dealing with economic health challenges and should not be overlooked within Q fever management and surveillance [6,13,14,15]. However, only 15 studies have recently been reviewed throughout the world, including South Africa, Cote d’Ivoire, Tanzania, Chad, Kenya, Lao People’s Democratic Republic (Laos), Belgium, Spain, the Netherlands, Australia, and the USA [6]. No ruminant or human monitoring is reported in France. With regard to animal health, it is only compulsory to report abortions within the framework of brucellosis surveillance [16]. Thus, the National Animal Epidemiological Surveillance Platform coordinates an observatory system for infectious abortions in ruminants, systematically including Q fever, in more than 20 departments in order to develop a feasible and harmonised reporting system. Previously, a large investigation into Q fever was conducted on ruminants in ten French departments between 2015 and 2017, showing that it is significantly enzootic (with seroprevalence ranging from 22.2% to 75.6%) [16]. The authors assumed that between 2.7% and 16.7% of abortive episodes in ruminants were linked to Q fever infection [16]. In terms of public health, the French National Reference Centre (NRC) of Q fever identifies sporadic human cases through its own diagnostic and clinical monitoring activities. Overall, the number of cases diagnosed or confirmed by the NRC between 2009 and 2022 was around 300 cases (ranging from 100–600) (personal communication from the NRC), but an accurate incidence rate cannot be assessed [12]. Since the creation of the French NRC in 1985, the partial picture obtained of human Q fever highlights how the challenge is intense within this disease [5].

The epidemiological contexts of Q fever in France remain unclear and have not yet been completely elucidated. To this end, we modestly attempted to identify the source as well as the epidemiological context of Q fever by using the One Health paradigm, focusing on human cases in the Hérault department in southeastern France.

## 2. Materials and Methods

### 2.1. Human Case Reports

In March 2021, a 50-year-old man living in the commune A (anonymized) in the south of the department of Hérault, between Montpellier and the Mediterranean Sea (south-eastern of France—Supplementary Data S1), was admitted for a consultation at one medical clinic, with one day-history of an influenza-like syndrome and a high fever (39 °C). The patient was COVID-19-negative, and no further symptoms were observed. Other than his sporting activity near his home, the patient had no history of travel during this period. He owned two dogs from the same region. The patient subsequently tested positive for antibodies against both I and II phases of *C. burnetii* using the indirect immunofluorescence assay at the Eurofins, Biomnis laboratory (Lyon, France). To investigate whether the origin of the Q fever was autochthonous, all Q fever cases confirmed by the same laboratory from this area in the last three years were retrieved. This revealed 12 other Q fever cases were serologically confirmed during the 2019–2022 period across four communes in the Hérault department, namely A, B, C, and D (Table 1). All these patients consulted due to respiratory symptoms.

### 2.2. Animal Sampling

Two field expeditions were organised to explore the potential origin(s) of Q fever in the Hérault department, during which we sought to sample domestic animals likely to be reservoirs of *C. burnetii* or sentinels of the infection (Supplementary Data S1). As the wildlife population (birds, rodents, foxes, wild boars, etc.) did not represent a classic source of Q fever, only domestic animals were targeted. First, in April 2022, blood and serum samples were obtained from a total of 233 animals, including 18 adult sheep, 113 dogs, and 102 horses, originating from four communes of the Hérault Department, in which the human patients had been observed. The second visit was performed in June 2022 and focused on the foci of Q fever detected in the sheep population. During the second expedition, all sheep from the investigated sheepfold were sampled. This included 21 animals (16 previously sampled adult sheep and three newly-sampled lambs). Seven goats, four horses, and thirty-nine dogs living close to the sheepfold were also included. All animals were subjected to blood and serum sampling. In addition, small ruminants (sheep and goats) were subjected to nasal, rectal, and vaginal swab sampling. All animals enrolled in the present study were not vaccinated against Q fever. Finally, environmental sampling of dust was performed using either a swab or a ready-to-use surface sampling kit (SodiBox, Névez, France), as described previously [17]. Briefly, dust was wiped from five different surfaces (i.e., manure, cobwebs, wooden boards, and concrete floors) in the sheepfold over a total length of 5 m (five times 1 m) each time. There is a single sheepfold and a pile of manure right next to it outside. While no goat or cattle breeding activity was found in the study areas, except in a butcher’s stall where oxen remain for a few weeks for fattening before being taken to the slaughterhouse. Faecal samples taken from a pile of cow manure were sampled as well. 

### 2.3. Serological Analysis

All serum samples were tested using a commercial Q fever ELISA kit (LSIVet Ruminant Serum/Milk, ThermoFisher Scientific, Illkirch-Graffenstaden, France). This was either used directly for testing ruminant sera or was adapted to screen samples from various mammal species by replacing the ruminant-specific conjugate with peroxidase-conjugated protein A/G, which has a strong affinity for various mammals, according to the customer (ThermoFisher Scientific—PierceTM). The optical density (OD) values were expressed in terms of “mean percentage of sample/positive” (S/P values): S/P value = (ODSample − ODNeg.control)/(ODPos.control − ODNeg.control) × 100. Positive and negative internal controls were included in each plate. Sensitivity and specificity performances were recently estimated at the threshold of 40% fixed by the supplier for ruminants [18]. In sheep, the sensitivity and specificity estimates were 53.8% 95 CI (43.3; 61.8) and 98.4% 95 CI (97.4; 99.3), respectively. The optimal seropositivity threshold of the multi-species ELISA test was defined at the S/P threshold of 40% for exploratory screening [19]. All serological analyses were performed at the Animal Q Fever Unit (ANSES Sophia Antipolis, French NRL for animal Q fever). Given the low sensitivity in sheep, true seroprevalence accounting for sensitivity and specificity was also estimated in a Bayesian framework (Thibaut Lurier, personal communication).

### 2.4. Geographical Plotting of Q Fever Cases

All human and animal Q fever cases were geographically plotted using the PowerBI software (V2.115.663.0) [20]. Mean values of wind data (i.e., speed, timing, and direction) recorded over the last three years by two meteorological stations converging the studied areas (Mediterranean airport station, 43 23′27.60 N, 3 57′49.37 E and Sète station: 43 24′19 N, 3 41′51 E) were used to generate the wind rose around the sheepfold. Exact binomial confidence interval and Charticulator tool [21] were used to calculate the Q fever seroprevalences among each species according to their distance from the sheepfold using 2 km distance schedules.

### 2.5. Molecular Analysis 

#### 2.5.1. Real-Time PCR Detection and Quantification of Bacterial Load

Blood samples from all serologically positive animals, as well as swab samples from small ruminants (sheep and goats), environmental samples, and faecal samples taken from a pile of cow manure, were subjected to a lysis step using 200 µL of buffer G2 supplemented with 25% proteinase K for 24 h at 56 °C prior to DNA extraction using the EZ1 DNA tissue kit (Qiagen, Courtaboeuf, France), following the manufacturer’s instructions.

Genomic DNA was subjected to molecular screening using the IS*1111* real-time qPCR system [10]. For bacterial quantification, we used the 10-fold serial dilution of 10^6^ concentrated synthesised plasmids as standard. The bacterial number for each sample was calculated by assuming that the number of *IS1111* elements of *C. burnetii* strain(s) in samples is similar to that in the genome of the Nine Mile strain of *C. burnetii* (20 copies) [22].

#### 2.5.2. Molecular Typing and Phylogenetic Analysis

All *C. burnetii*-positive samples by qPCR assay were subjected to multispacer sequence typing (MST), as described elsewhere [23]. Briefly, each sample was subjected to spacer-specific amplification using primers sets listed in Supplementary Table S1. PCR reaction was carried out in a total volume of 50 µL, consisting of 25 µL of AmpliTaq Gold master mix (Thermo Fisher Scientific, Waltham, MA, USA), 18 µL of ultra-purified water DNAse-RNAse free, 1 µL of each primer, and 5 µL of genomic DNA. PCR reactions with all systems were run under the following protocol: incubation steps at 95 °C for 15 min, 40 cycles of 1 min at 95 °C, 30 s for the annealing at a custom melting temperature for each assay (Table S1), and elongation for 45 s at 72 °C with a final extension for 5 min at 72 °C. All PCR amplifications were carried out in a thermal cycler (Applied Biosystem, Paris, France), as described elsewhere. DNA amplicons were purified using NucleoFast 96 PCR plates (Macherey Nagel EURL, Hoerdt, France) prior to the sequencing reaction with the Nanopore sequencer.

##### MinION Library Preparation and Multiplexed Nanopore Sequencing

The sequencing library was prepared using the 1D Native barcoding genomic DNA protocol with EXP-NBD104 and SQK-LSK110 kits (Oxford Nanopore Technologies, Oxford, UK). PCR products were first purified using the filter plate Millipore NucleoFast 96 PCR kit according to the manufacturer’s recommendations (Macherey Nagel, Düren, Germany). Approximately 230 fmol of purified amplification product was subjected to DNA repair and end-prep using a NEBNext DNA repair mix and NEBNext Ultra II End Repair/dA-Tailing Module (New England Biolabs, Ipswich, MA, USA). The library preparation included two ligation steps. In the first step, multiplexing barcodes were ligated to 500 ng of each of the processed amplification products from each sample, using T4 Ligase (New England Biolabs). Equal molarities of the barcoded amplicons were pooled together, and 630 ng of the pooled sample was subjected to MinION adaptor ligation according to the protocol. Each step was followed by DNA purification with AMPure XP Beads (AXP) (Oxford Nanopore Technologies). The sequencing run was performed on the MinION MK1C according to the instructions from Oxford Nanopore Technologies (ONT). The library was loaded to the Nanopore MinION Spot-on flow cell (R10.4.1) and sequenced until reaching ~2.5 Gb (~2.7 M reads). Base calling and barcode demultiplexing were performed automatically by the MinKnow programme. Raw reads were obtained in FAST5 and FASTQ formats, and “pass” quality reads were subjected to further analysis.

##### Data Analysis and Multi-Loci Sequence Typing

Demultiplexed reads representing each sample were subjected individually to a second demultiplexing to separate reads amplicons based on primer sequences of each spacer within the nanoMLST scripts [24]. Adapters were trimmed with qcat (https://github.com/nanoporetech/qcat (accessed on 27 March 2023)), and primers were removed using Cutadapt and default parameters with 10% error tolerance [25]. Read quality was estimated using NanoPlot [26]. Sequences were filtered based on read quality (-min_qual_mean 10), and read length was restricted (-min_len X -max_len Y 250–800 bp) using prinseq-lite V0.20.4 [26]. In order to increase the accuracy of the Nanopore MinION reads [27,28], a consensus sequence for each spacer from each sample was generated using ONTrack pipeline (https://github.com/MaestSi/ONTrack (accessed on 27 March 2023)) according to Maestri et al. [28]. Consensus sequences of all spacers (i.e., *Cox2, Cox5, Cox18, Cox20, Cox22, Cox37, Cox51, Cox56, Cox57,* and *Cox61*) were first compared against the MST database available at https://ifr48.timone.univ-mrs.fr/mst/coxiella_burnetii/blast.html (accessed on February 2022). Phylogenetic analysis on the concatenated spacers was performed using the maximum likelihood model. Briefly, MST sequences were aligned against all available MS types of *C. burnetii* (https://ifr48.timone.univ-mrs.fr/mst/coxiella_burnetii/blast.html (accessed on 27 March 2023)) using mafft [29] prior sequence concatenation within Seaview [30]. The ML phylogram was generated using IQ-TREE [31] under 10,000 ultra-fast bootstrap (UFBoot) replicates and the HKY+F+R2 model according to Modelfinder [32] (implemented as functionality of IQ-TREE). Tree editing was performed using iTOL v5 software [33].

## 3. Results

### 3.1. Human Q Fever Cases

Chronologically, 12 patients were diagnosed before our investigation, one of whom, from commune A, was the alert launcher, diagnosed in March 2021. One patient from commune B was diagnosed during the current investigation in June 2022.

A total of 13 patients (women between the ages of 51 and 75, n = 5, and men aged between 37 and 70, n = 8) from four communes (A, B, C, and D in the Hérault department) were serologically confirmed positive for Q fever disease during the 2019–2022 period (Table 1). The average prevalence was 4.3 cases per year. With a total population of 23,000 in the four municipalities (according to the official census of 2020), the current prevalence of human Q fever is 19 cases per 100,000 inhabitants in this area.

### 3.2. Serological Screening of Q Fever from Animals

The results of serological analyses of Q fever are listed in Table 2. In total, 13.4% (95 CI (12.8; 14)) of the 284 animals tested were positive for anti-*C. burnetii* antibodies. This included ten sheep, nineteen dogs, and nine horses. All goats (n = 7) belonging to one flock were serologically negative for Q fever (Table 2).

In terms of seroprevalence, the highest rate was found in the population of sheep housed in the same sheepfold, at 47.6% (95 CI (45.5; 49.7)) (which corresponds to a true seroprevalence of 60% 95 CI (33; 88) (Thibaut Lurier, personal communication), followed by dogs with 12.6% (95 CI (11.8; 13.4)) and horses with 8.49% (95 CI (7.5; 9.4)). All positive adult sheep detected in the first expedition (April 2022) were confirmed in the second expedition (June 2022) except for two animals that were negative, while none of the three lambs sampled in June was positive (Table 2). Antibody levels up to 80% S/P were maintained in seven animals.

### 3.3. Real-Time PCR Detection and Bacterial Quantification

The detailed results of qPCR screening and quantification are listed in Table 3 and Table 4. DNA of *C. burnetii* was detected in the nasal swabs of five adult sheep and in only one vaginal swab. At the same time, none of the rectal swabs was positive by IS*1111* qPCR assays. All qPCR-positive samples originated from animals found to be serologically positive. Based on IS*1111* qPCR, bacterial load ranged between (10 × 10^3^–10 × 10^6^) bacteria per mL of sample in nasal swabs and (< 1000) bacteria per mL in the positive vaginal swab. Finally, *C. burnetii* DNA was detected in all environmental swabs by the IS*1111* qPCR (Table 4).

### 3.4. Mapping Human and Animal Q Fever Cases

**Figure 1:**
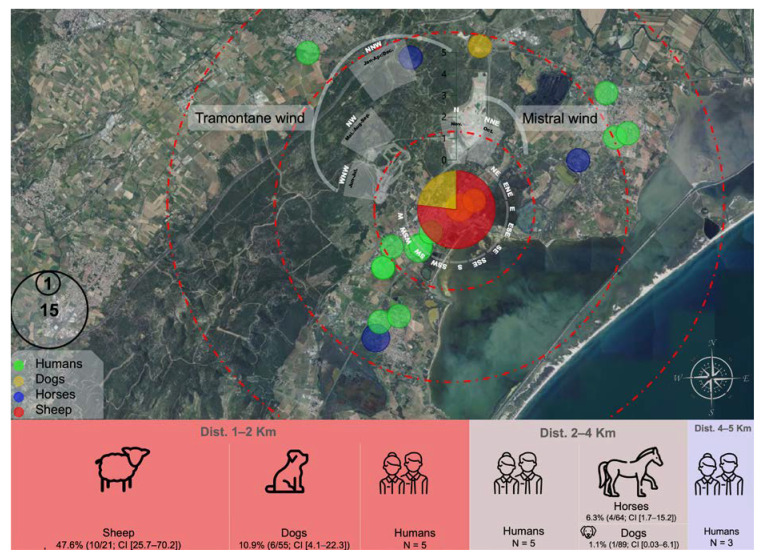
Geographical plotting of human and animal Q fever cases detected in the four French communes (Hérault department). Circles are colour-coded and size-dependent according to the host and number of cases. The wind rose around the sheepfold indicates the name, intensity (wind speed), direction, and annual active periods over the past three years. Geographical locations of both human and animal cases are approximative and do not reflect the exact location of Q fever cases in order to maintain confidentiality. Number of human cases (N), seroprevalence as well as 95 CI are provided for each sampled population within 2 Km distance schedules from the sheepfold.

To better understand whether the sheepfold was at the origin of human and animal Q fever cases, all cases were mapped, and their exposure to the annual wind activity (Tramontane and Mistral winds) was indicated (Figure 1).

Q fever seroprevalence was concentrated within the first 2 km perimeter around this sheepfold with a range of 29% (95 CI (27.9; 30.1)) among the 86 animals sampled, followed by 7.14% (95 CI (6.4; 7.9)) among the 154 animals sampled, and 4.54% (95 CI (3; 6)) among the 44 animals sampled within the first, second and third 2 km perimeter around the sheepfold, respectively (Table 5, Figure 1).

The homes of human cases were concentrated around the sheepfold, with five cases within each of the first and the second 2 km perimeters and three cases within a perimeter of more than 4 km (Table 1 and Figure 1).

### 3.5. C. burnetii Genotyping

**Figure 2:**
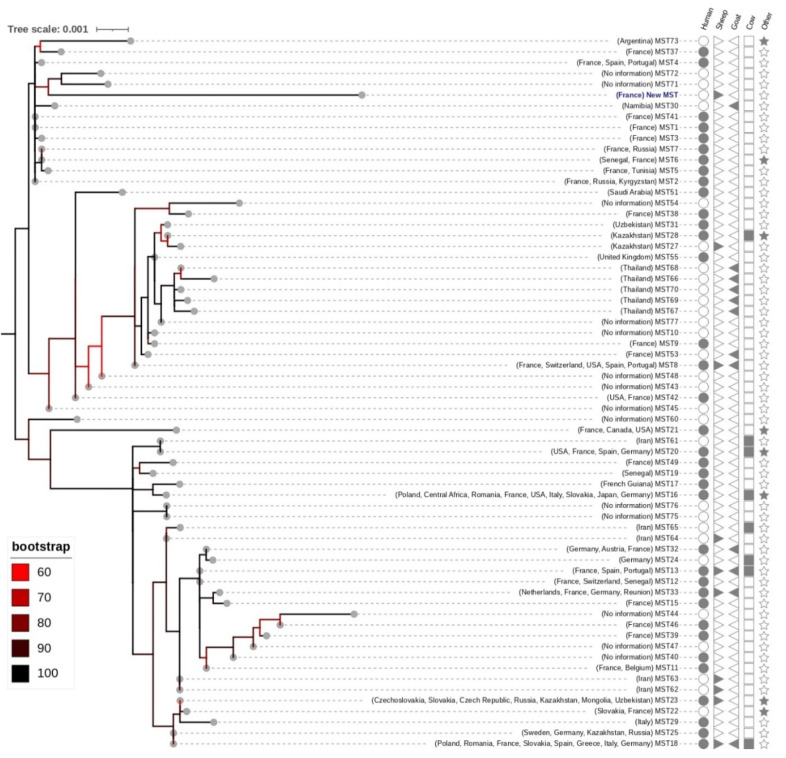
ML phylogeny showing the position of the new *C. burnetii* genotype obtained in the present study (blue label) among all available genotypes identified so far. The tree was generated using the ML method under 10,000 bootstrap replications. The tree involved 66 sequences type of 4,638 bp with 4% of informative sites. Geographical origin, host, and MS Id are indicated at each node. Branches are colour-coded according to bootstrap value. The tree is rooted at the midpoint.

All the target spacers were amplified from three out of the five qPCR-positive samples (OVG3, OVG4, and OVG16). Accordingly, consensus sequences of the amplified DNA of the ten spacers were successfully obtained for the three sequenced samples and were identical to one another for these samples. The MST blast indicated a new sequence type for each of the obtained spacers. Likewise, ML phylogeny indicated that the *C. burnetii* genotype from the present study clustered in a distinct group with MST71 and MST72 (Figure 2). 

## 4. Discussion

Concern among general practitioners about potentially excessive human cases of Q fever prompted an investigation into an area covering four municipalities in the Hérault department from the south of France. The present study, based on the One Health approach, focused on describing the epidemiological pressure caused by *C. burnetii* among animals and humans. 

The antibody levels detected in each animal in the foldsheep were comparable between the first and the second sampling point, which may be due to the shortness of the period between the two samples (3 months). The apparent seroprevalence estimated in sheep (47.6% (95 CI (45.5; 49.7)), n = 21) aligns with previous results reported in several French departments [16], confirming the continuous risk of exposure and the role of domestic ruminants in Q fever dissemination in France. By means of comparison, the within-herd seroprevalences were evaluated at the department level in France using the same ELISA kit, and the average among seropositive herds ranged from 10.2% to 56.2% for goats, 7.0% to 36.5% for sheep and 12.2% to 30.5% for cattle, showing high variability [34]. Since the sensitivity of the ELISA kit used has recently been evaluated to be low in sheep (Se = 53.8% 95 CI (43.3; 61.8)), the apparent seroprevalence might underestimate the true proportion of seropositive sheep (corrected to 60% 95 CI (33; 88)). The high true seroprevalence detected in sheep with high individual antibody levels suggests active shedding and circulation of *C. burnetii* within the flock of sheep. Indeed, high individual antibody levels were measured for eight ewes (44.5% (95 CI (42.2; 46.8)) with a level greater than 80% S/P, n = 18). The pattern observed according to age indicated that six young ewes (between one and three years old) had strong antibody levels (75.0% (95 CI (71.5; 78.5)), n = 8) compared to two out of seven older ewes (28.6% (95 CI (24.9; 32.3))), suggesting that *C. burnetii* bacteria circulated during the last three years.

Regarding bacterial shedding, the flock was tested in summer (June), outside the lambing season. To maximise the detection of bacterial discharge, two shedding routes were tested, as the concomitancy of vaginal and faecal shedding routes would be scarce, and shedding in vaginal mucus may be very short in duration after abortion or lambing [4,34]. Furthermore, litter and manure, where placentas and birth fluids are also deposited from *C.-burnetii*-positive sheep and goat farms are a major source of human Q fever infections [35]. The absence of *C. burnetti* DNA in almost all (except one vaginal swab) vaginal and rectal swabs herein tested suggests that bacterial shedding was negligible at the time of sampling. A persistently high antibody level found in seven animals without shedding raises questions. The relationship between serological response and bacterial shedding was not clear between infected animals, but most results supported the following infection patterns. A weak or negative antibody response may correspond to exposure to the pathogen either in the past (the response decreases) or recently (the response increases). Individuals may also be seronegative and shedding due to being in the early stages of infection [19,36,37]. Individuals with high antibody levels would be mostly continuous and strong shedders, and the others would be weak or intermittent shedders and non-shedders in goats [36,38], cattle [39], and sheep [40,41,42]. Therefore, the highly seropositive response of non-shedder ewes in this study might result from a continuous immune boost due to dead bacteria or a micro-dose of viable bacteria that was regularly aerosolised in the environment. We cannot rule out the possibility that this occurred due to a localised persistent infection among ruminants not causing vaginal or faecal bacterial excretion, as discussed elsewhere [40,43].

Unlike bacterial shedding, the PCR results on environmental dust samples were positive. The results showed that the highest concentration of environmental *C. burnetii* was found in sheep birthing areas. Moreover, *C. burnetii* DNA was also detected in nasal swabs, underlining the dust results. Testing the air inhaled by the sheep represents a novel approach to estimating environmental contamination [34,44]. Taken as a whole, this field data is insufficient to fully understand shedding dynamics, i.e., to know when the shedding started, whether it was on a large scale, how long it lasted, and whether it could be reactivated in the future. Several studies have provided temporal data on bacterial excretion by sheep during an “abortion wave” and for successive lambing seasons [42,44,45]. The infection can remain active for over five years in some flocks [45] and could be maintained for 10 years, probably due to periodical reinfections [46]. Here, no history of abortion waves attributed to Q fever had been reported for the sheep flock, although it is also common to encounter asymptomatic (or unnoticed subclinical) flocks/herds where bacteria are being shed. In such cases, the number of organisms being shed is typically much lower than that seen in aborting flocks/herds. In the current study, the fact that few or no bacteria were shed by ewes, while a significant load was found in dust, suggests that the shedding likely occurred during the previous lambing period(s), leading to cumulative contamination of the environment, and then decreasing until extinction. The absence of historical shedding monitoring does not allow us to conclude that shedding has taken place since 2019 in this farm, but the analysis of serology results according to age groups supports this hypothesis. Conversely, the fact that yearlings were seronegative suggests that exposure to contaminated dust did not take place in recent months.

Investigations conducted on other animals in the area revealed the presence of anti-*C. burnetii* antibodies in 8.49% (95 CI (7.5; 9.4)) and 12.6% (95 CI (11.8; 13.4)) of horses and dogs tested, respectively. These two species are considered sentinel indicators of the infection [47,48]. These seroprevalence rates may reflect the airborne dissemination of *C. burnetii* and a spatial exposure level from the Q fever-positive sheepfold. This hypothesis could be supported by the fact that the wind mainly blows in two directions, extending the dissemination of *C. burnetii* bacteria towards areas to the east–southeast and west–southwest from the sheepfold. The involvement of wind in Q fever dissemination has already been reported [8,49]. In France, wind was suggested to have played a role in the airborne transmission of *C. burnetii* in previous outbreaks in several regions [2,50,51]. Furthermore, the dissemination of Q fever through other animals and humans was limited to a 6 km perimeter around the sheepfold. The same 4–6 km perimeter has previously been reported regarding wind dissemination of Q fever [2,50,51].

In the present study, the human cases of Q fever that were detected are likely to be due to their presence around the sheepfold (living near or walking along the tourist road, which is to the east–southeast of the sheepfold, thus increasing their exposure to the wind) or to them having been in direct contact with infected animals or the contaminated manure. The number of cases was too low to statistically assess the seasonality of the cases (three in 2019 from June to November, six cases from June to September 2020, three cases from March to December 2021, and one case in June 2022). Given the incubation period of around one month, a trend can be observed, with a period at risk of infection in spring and summer, which is in accordance with the findings of other studies [7], suggesting that viable *C. burnetii* were more widespread during these periods of post-parturition and manure spreading.

To sum up, several elements support the hypothesis that this sheepfold may be the source of some cases among human subjects living in the area: 1/ a significant proportion of infected animals (ewes, dogs) and bacterial contamination accumulating in the environment; 2/ strong winds in this area; 3/ proximity to a through road and the homes of the human cases; 4/ other animals found to be seropositive (dogs, horses) in the farm surroundings. Nevertheless, this hypothesis is questionable and reveals several shortcomings when it comes to determining the origin of human cases.

Firstly, the evolution of the infection in this farm may have been self-limited in time as the lambs were seronegative, and the proportion of shedders seemed minimal. The epidemiological cycle was also difficult to decipher as the survey was carried out over a short period in 2022, which, unfortunately, did not allow us to observe the dynamics of distribution over the previous three years among the various animal populations. Horses and dogs were found to be seropositive for Q fever. Though few goats (n = 7) were herein tested, which may represent a limitation of the study, the complete absence of positive individuals among them remains unclear as the area is supposed to be at risk. The seronegative results of *C. burnetti* on the ram in the sheep flock suggest that sexual transmission of *C. burnetti* is minor, which may be due to the absence of vaginal contamination during the mating. Some animals were not tested: a butcher’s stall, some small ruminants, and pets could not be included. Secondly, the results of environmental contamination contain complex information.

The *C. burnetii* contamination loads found in dust swabs from the sheepfold were considered non-negligible in comparison to previous reports from France and other countries [17,34,50,52]. Positive samples ranging from 10^5^ to 10^7^ genome equivalents per swab were consistent with relatively high loads in asymptomatic sheep flocks in France using the same standardised protocol for surface samples [17]. Nevertheless, the qPCR quantified *C. burnetii* DNA in dust might correspond to degraded bacteria or to viable bacteria. Studies have shown that spore forms of *C. burnetii* can be highly resistant [34,53,54,55,56,57,58], but monitoring studies are lacking on a long-lasting persistence assessing experimental or field conditions. Definitive evidence is also lacking on spore resistance capacity, which may vary drastically amongst different *C. burnetii* strains. Novel, simple, and rapid testing is urgently needed to assess viable bacteria load and the duration of persistence in ruminant housing/birthing areas and other environments. Classical isolation techniques based on animal models are laborious to apply on a large scale and are not recommended for ethical and technical reasons [45,52,59,60,61]. Further long-term investigations are also needed to evaluate any association with the risk of infection. The aerial infectious dose is still under discussion [62,63,64]. Presumably, bacterial concentrations in aerosols need to be high for substantial transmission via the respiratory route [65]. Furthermore, the effective transmission of low atmospheric concentrations seems to require directed flow (i.e., strong wind, air conditioning, proximity to a heliport, and resuspension activities such as spreading manure or outdoor wool shearing) [65]. In addition to the need for favourable transmission conditions, a large variation in the infectious dose could be influenced by factors exhibited by the strain involved and, at least, by the host. The human time–dose response model proposed recently [63], which takes into account the infectious dose and incubation period, is likely to be modulated according to population categories. Basically, *C. burnetii* is a work-related disease whereby workers in contaminated breeding areas are the most exposed, as reported previously [50]. The proportion of Q fever-seropositive individuals is often high, but the people who develop the disease rarely live and work in rural areas because those who do appear to be able to acquire natural immunity. The number of cases is, therefore, probably partly controlled for people who have lived and worked in agricultural areas for many years [7]. Thus, even if the bacterial loads measured in the dust samples are relatively high and viable, firm conclusions on the implications of this for human health can still not be drawn. More studies are needed to decipher the diversity of environmental contamination configurations and their links to the incidence of human cases [52,63]. Dust swabs from surfaces can be a useful and affordable tool for detecting *C.-burnetii*-positive livestock [17], but reliable contamination thresholds indicating the risk of a Q fever epidemic are still difficult to establish.

Finally, it is difficult to confirm that the sheepfold was at the origin of the human cases because people may have been infected before the sheepfold was infected or may have been infected elsewhere, including in other regions, while traveling or taking part in other activities. Significantly, the qPCR data produced over the last decade represents good progress towards understanding the quantity and spatial distribution of environmental contamination, although epidemiological analysis needs to be combined with the use of genotyping/genomotyping methods [66]. The main prerequisite for the One Health approach is to obtain and compare patients’ strains with those of the suspected source(s). Extensive investigations of circulating genotypes have been carried out in several countries, including France [67]. Work is also in progress to analyse the pangenome and to share a central platform in the form of a genotyping database [68]. Innovations are still required for genotyping and whole genome sequencing methods in order to gain the ability to distinguish between several strains in dust samples and to allow sufficiently accurate tracing to manage outbreaks. Here, the implementation of Nanopore sequencing with dual barcoding analysis yielded the identification of a new *C. burnetti* genotype from three out of five qPCR-positive nasal swab samples. These findings confirm the utility of Nanopore barcoding in multi-target gene typing, as suggested elsewhere [24]. However, it depends on the number of PCR amplicons from samples with a qPCR threshold of less than 32. Due to the tandem repeat of the IS*1111*, the qPCR detection remains more sensitive compared to standard PCR, as previously reported for the MLVA/VNTR typing [69]. Phylogenetically, the new genotype of *C. burnetii* is closely related to the MS71-72 genotypes. However, no information was available on these genotypes from the MST database (https://ifr48.timone.univ-mrs.fr/mst/coxiella_burnetii/blast.html (accessed on 24 March 2023)). One perspective of this study will be to determine whether this genotype is particularly involved in the occurrence of human cases in the south of France. 

In humans, Q fever can lead to clinical developments several years after infection, including severe cases (endocarditis, Q fever fatigue syndrome). Diagnosis is difficult due to the range of various non-specific symptoms. Antibiotic treatment is often lengthy once the infection is established, and early diagnosis is encouraged. However, one major difficulty that remains is how to determine when a number of cases are unusual in a given sector. Currently, it is not mandatory in France to notify the authorities of human Q fever cases. As a result, no solid background data are available at the department level, and human Q fever cases are likely to be underestimated. For example, 92 human cases of Q fever were reported in 2021 by French health authorities, which represents 0.13/100,000 inhabitants [12]. Nonetheless, the present study highlighted a prevalence of 19/100,000 inhabitants in the investigated areas. These figures suggest either a hyperendemicity in the studied area or a high underestimation of the impact of Q fever in France. In addition, a variety of commercially available tests are used, and the concordance rate of their results has not yet been assessed. A review of data from commercial laboratories is also necessary to ensure that serological titre patterns meet diagnostic criteria [70]. It would be relevant to harmonise and centralise case data from all medical analysis laboratories offering Q fever testing and to spatio-temporal patterns.

## 5. Conclusions

These results highlight the importance of implementing epidemiological surveillance of Q fever in France in order to improve the detection and management of outbreaks [8]. They also reveal the high endemicity in certain sectors [71,72]. This encourages using an economic monitoring tool for surface samples and the development of innovative tests for viability and genetic characterisations applicable to environmental matrices to gain deeper insight into the various transmission scenarios. 

## Figures and Tables

**Table 1:** Geographical distribution of human cases of Q fever diagnosed between 2019 and 2022 in four French communes in the Hérault department.

Date	Patient Id	Gender	Age	Distance from the Sheepfold (m)
June 2019	1	M	30–40	4220
September 2019	2	M	60–70	4850
November 2019	3	F	70–80	2710
May 2020	4	M	50–60	2100
June 2020	5	M	40–50	3920
June 2020	6	M	40–50	4200
July 2020	7	F	50–60	2100
September 2020	8	M	60–70	1130
December 2020	9	F	60–70	766
March 2021	10	M	50–60	1210
May 2021	11	F	60–70	1670
December 2021	12	F	50–60	1210
June 2022	13	F	50–60	3060

**Table 2:** Distribution of Q fever seroprevalence according to animal species and their distance from the sheepfold.

Origin of Animals	Location (Commune)	Distance from the Sheepfold (m)		Sheep			Goats		Dogs			Horses			All Animals		
			No.	Positive	%	No.	Positive	%	No.	Positive	%	No.	Positive	%	No.	Positive	%
Sheepfold	A	0	21	10	47.6				3	3	100				24	13	54.1
Kennel 1	A	80	-		-				6	4	66.6				6	4	66.6
Owner 1	A	445	-		-	6	0	0	2	1	50	4	1	25	12	2	16.6
Kennel 2	A	548	-		-				4	0	0				4	0	0
Owner 2	A	989	-		-				1	0	0				1	0	0
Kennel 3	A	1140	-		-				34	4	11.7				34	4	11.7
Owner 3	B	1210	-		-				2	1	50				2	1	50
Owner 4	A	1350	-		-				1	0	0				1	0	0
Owner 5	A	1440	-		-				1	1	100				1	1	100
Owner 6	A	1590	-		-				1	0	0				1	0	0
Stable 1	C	2960	-		-	1	0	0	4	0	0	14	1	7	19	1	5.2
Kennel 4	B	3240	-		-				10	0	0				10	0	0
Stable 2	B	3400	-			-						10	3	30	10	3	30
Stable 3	D	3500	-			-						40	2	5	40	2	5
Kennel 5	C	3730	-			-			70	5	7				70	5	7
Kennel 6	B	3760	-			-			5	0	0				5	0	0
Owner 7	B	4250	-			-			3	0	0				3	0	0
Owner 8	B	4820	-			-			2	0	0				2	0	0
Stable 4	B	5080	-			-			1	0	0	22	1	4.5	23	1	4.43
Stable 5	D	6330	-			-						16	1	6.2	16	1	6.2
TOTAL			21	10	47.6	7	0	0	150	19	12.6	106	9	8.49	284	38	13.38

**Table 3:** Anti-*C. burnetii* antibodies and real-time quantitative PCR results on sheep samples.

			Serological Analyses (PrioCHECK ^TM^ Q Fever)				Molecular Analysis Using IS*1111* qPCR						
Sheep Number	Gender	Age (Years)	First Screening: 7 April 2022		Second Screening: 7 June 2022		Nasal Swabs			Vaginal Swabs			Rectal Swabs
			Interp.	S/P%	Interp.	S/P%	Ct Value	IS Copy Number/ Reaction	Genome Equivalent Copy Number/mL	Ct Value	IS Copy Number/ Reaction	Genome Equivalent Copy Number/mL	Ct Value
OVG1	F	3	POS++	177	POS++	176	ND			ND			ND
OVG2	F	3	NEG	26.2	NEG	19.4	ND			ND			ND
OVG3	F	7	POS++	185.6	POS++	188.9	26.6	75,600	756,000	ND			ND
OVG4	F	3	NEG	6.2	NEG	6.6	31.3	3,090	31,000	ND			ND
OVG5	F	8	POS++	47	NEG	35.6	34.3	422	4200	ND			ND
OVG6	F	9	POS++	46.4	NEG	31.6	ND			ND			ND
OVG7	F	3	POS++	113	POS++	91.3	ND			ND			ND
OVG8	F	9	NEG	14.7	NEG	16	ND			ND			ND
OVG9	F	1	POS+++	232.1	POS+++	250.9	ND			ND			ND
OVG10	F	8	NEG	17.6	NEG	17.3	ND			ND			ND
OVG11	F	3	NEG	24.9									
OVG12	F	3	NEG	10.8	NEG	5.6	ND			36.94	70.9	800	ND
OVG13	F	3	POS+++	205.1	POS+++	175.8	34.3	407	4000	ND			ND
OVG14	F	3	NEG	15									
OVG15	F	3	POS+	70.8	POS+	50.8	ND			ND			ND
OVG16	M	8	NEG	9.5	NEG	9.1	29.4	11,100	111,000				ND
OVG17	F	3	POS++	190.4	POS+++	288.5	ND			ND			ND
OVG18	F	6	POS+	82.7	POS+	99.2	ND			ND			ND
OVM19	M	3 months			NEG	3.30	ND						ND
OVM20	M				NEG	2.70	ND						ND
OVM21	F				NEG	3	ND			ND			ND

POS: positive; NEG: negative; ND: not detected; +: low; ++: moderate; +++: hight. The calculated bacterial load using IS copy number/reaction is an estimation as the number of IS copies per genome varies according to *C. burnetii* strains. For instance, one genome of the reference Nine Mile strain includes 20 copies of IS*1111,* and the number of IS*1111* is known to vary between 7 and 110 copies according to strains [22].

**Table 4:** Result of real-time quantitative PCR testing on environmental samples.

Sample Type	Environmental Swabs in the Sheepfold	IS*1111* qPCR		
		Ct Value	Genome Equivalent Copy Number /mL	Class per Sample
Swab	Manure	35.57	170	[<1000 bact.]
	Cobweb	26.89	61,320	[10 × 10^4^–10 × 10^5^]
	Wooden board	26.07	106,000	[10 × 10^4^–10 × 10^5^]
	Concrete floor	29.19	13,000	[10 × 10^4^–10 × 10^5^]
Cloth (SodiBox^®^)	Watering hole	33.78	39,000	[10 × 10^5^–10 × 10^7^]
	Left feeder	31.25	222,000	[10 × 10^5^–10 × 10^7^]
	Right feeder	30.02	521,000	[10 × 10^5^–10 × 10^7^]

**Table 5:** Seroprevalence in animals and human cases of Q fever around the sheepfold according to 2 km perimeters.

Contamination Area	Q Fever Seroprevalence in Animal			Human Cases
	Number of Animals	Number of Positive Cases	%	
2 km radius around the sheepfold	86	25	29	5
Between 2 and 4 km around the sheepfold	154	11	7.14	5
Between 4 and 6 km around the sheepfold	44	2	4.54	3
TOTAL	284	38	13.38	13

## Data Availability

Code: consensus sequences and Raw reads are available as a GitHub repository (https://github.com/YLdz-SM/Nanopore-MinION-with-a-dual-barcoding-for-MS-Typing-of-C.-burnetti).

## Supplementary Materials

The following are available online at https://www.mdpi.com/article/10.3390/microorganisms11041016/s1; Figure S1: Geographical map showing the location of the outbreak. Table S1: Primer sets and PCR conditions used in the present study according to [69].
