# Supplementary figures and images for: Tracking the Source of Human Q Fever from a Southern French Village: Sentinel Animals and Environmental Reservoir
Source: Microorganisms. 2023 Apr 13;11(4):1016. doi: 10.3390/microorganisms11041016 (PMC10142994; doi:10.3390/microorganisms11041016)

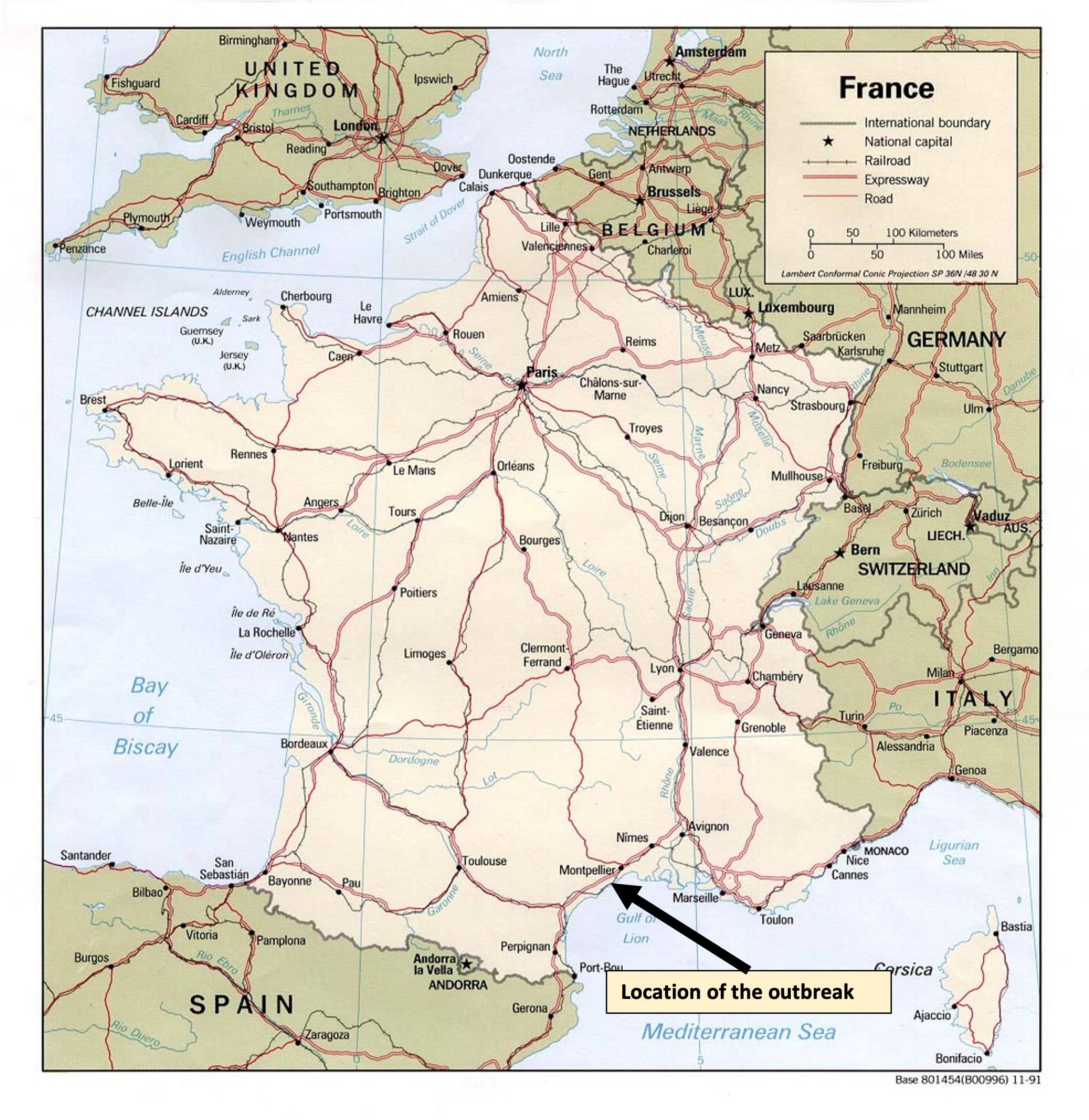

Supplement: Supplementary file 1 [file microorganisms-11-01016-s001.zip › Figure S1.png]
